# Supplementary material for: Genetic assessment of farmed Oreochromis mossambicus populations in South Africa
Source: PeerJ. 2025 Apr 14;13:e18877. doi: 10.7717/peerj.18877 (PMC12005181; doi:10.7717/peerj.18877)
Supplement: Supplemental Information 1 [file peerj-13-18877-s001.docx]

**Supplementary Table 1:** Pairwise Nei’s genetic distance estimates for the four farmed and eight wild *O. mossambicus* populations in Mpumalanga and KwaZulu-Natal. (Note: Zini farm = Zini Fish Farm; UniZulu ponds = University of Zululand; Fresca farm= Fresca Fisheries Farm; Pieter Dam = Pieter Vorster Dam).

| Population | Zini farm | UniZulu ponds | uMphafa ponds | Mfolozi | Mhlathuze | Matigulu | Thukela | Mvoti | Fresca Farm | Komati | Pieter Dam | Loskop Dam |
| --- | --- | --- | --- | --- | --- | --- | --- | --- | --- | --- | --- | --- |
| Zini farm | 0.00 |  |  |  |  |  |  |  |  |  |  |  |
| UniZulu ponds | 0.12 | 0.00 |  |  |  |  |  |  |  |  |  |  |
| uMphafa ponds | 1.15 | 1.23 | 0.00 |  |  |  |  |  |  |  |  |  |
| Mfolozi | 0.22 | 0.16 | 0.91 | 0.00 |  |  |  |  |  |  |  |  |
| Mhlathuze | 0.09 | 0.06 | 0.98 | 0.12 | 0.00 |  |  |  |  |  |  |  |
| Matigulu | 0.16 | 0.27 | 1.43 | 0.21 | 0.24 | 0.00 |  |  |  |  |  |  |
| Thukela | 0.33 | 0.31 | 0.62 | 0.20 | 0.20 | 0.47 | 0.00 |  |  |  |  |  |
| Mvoti | 0.24 | 0.30 | 0.80 | 0.34 | 0.20 | 0.30 | 0.17 | 0.00 |  |  |  |  |
| Fresca Farm | 0.34 | 0.24 | 0.78 | 0.24 | 0.33 | 0.44 | 0.38 | 0.52 | 0.00 |  |  |  |
| Komati | 0.41 | 0.48 | 0.74 | 0.44 | 0.45 | 0.45 | 0.37 | 0.44 | 0.30 | 0.00 |  |  |
| Pieter Dam | 0.49 | 0.53 | 1.32 | 0.49 | 0.56 | 0.66 | 0.82 | 0.98 | 0.54 | 0.65 | 0.00 |  |
| Loskop Dam | 0.41 | 0.59 | 0.81 | 0.61 | 0.60 | 0.51 | 0.55 | 0.52 | 0.31 | 0.13 | 0.93 | 0.00 |
