# Supplementary material for: Genetic assessment of farmed Oreochromis mossambicus populations in South Africa
Source: PeerJ. 2025 Apr 14;13:e18877. doi: 10.7717/peerj.18877 (PMC12005181; doi:10.7717/peerj.18877)
Supplement: Supplemental Information 2 [file peerj-13-18877-s002.docx]

**Supplementary Table 2:** Estimates of ENA-corrected pairwise F*_ST_*-values and probability (P-values) of the genetic differences for the four farmed and eight wild *O. mossambicus* from Mpumalanga and KwaZulu-Natal. (Note: Zini farm = Zini Fish Farm; UniZulu ponds = University of Zululand; Fresca farm= Fresca Fisheries Farm; Pieter Dam = Pieter Vorster Dam).

| Population | Zini farm | UniZulu ponds | uMphafa ponds | Mfolozi | Mhlathuze | Matigulu | Thukela | Mvoti | Fresca Farm | Komati | Pieter Dam | Loskop Dam |
| --- | --- | --- | --- | --- | --- | --- | --- | --- | --- | --- | --- | --- |
| Zini farm |  | 0.0008^*^ | 0.0008^*^ | 0.0015^NS^ | 0.0053^NS^ | 0.0008^*^ | 0.0008^*^ | 0.0008^*^ | 0.0008^*^ | 0.0008^*^ | 0.0008^*^ | 0.0008^*^ |
| UniZulu ponds | 0.09 |  | 0.0008^*^ | 0.0008^*^ | 0.0008^*^ | 0.0008^*^ | 0.0008^*^ | 0.0008^*^ | 0.0008^*^ | 0.0008^*^ | 0.0008^*^ | 0.0008^*^ |
| uMphafa ponds | **0.36** | **0.42** |  | 0.0008^*^ | 0.0008^*^ | 0.0008^*^ | 0.0008^*^ | 0.0008^*^ | 0.0008^*^ | 0.0008^*^ | 0.0008^*^ | 0.0008^*^ |
| Mfolozi | 0.12 | 0.09 | **0.36** |  | 0.0008^*^ | 0.0015^NS^ | 0.0008^*^ | 0.0008^*^ | 0.0015^NS^ | 0.0030^NS^ | 0.0008^*^ | 0.0008^*^ |
| Mhlathuze | 0.04 | 0.04 | **0.34** | 0.08 |  | 0.0008^*^ | 0.0008^*^ | 0.0008^*^ | 0.0008^*^ | 0.0008^*^ | 0.0008^*^ | 0.0008^*^ |
| Matigulu | 0.11 | 0.19 | **0.45** | 0.18 | 0.15 |  | 0.0008^*^ | 0.0008^*^ | 0.0008^*^ | 0.0008^*^ | 0.0008^*^ | 0.0008^*^ |
| Thukela | 0.15 | 0.18 | **0.27** | 0.13 | 0.11 | 0.24 |  | 0.0008^*^ | 0.0008^*^ | 0.0008^*^ | 0.0008^*^ | 0.0008^*^ |
| Mvoti | 0.12 | 0.18 | **0.33** | 0.20 | 0.11 | 0.18 | 0.11 |  | 0.0008^*^ | 0.0008^*^ | 0.0008^*^ | 0.0008^*^ |
| Fresca Farm | 0.15 | 0.14 | **0.30** | 0.13 | 0.15 | 0.24 | 0.16 | 0.23 |  | 0.0023^NS^ | 0.0008^*^ | 0.0008^*^ |
| Komati | 0.13 | 0.20 | **0.26** | 0.18 | 0.14 | 0.19 | 0.13 | 0.16 | 0.11 |  | 0.0008^*^ | 0.0258^NS^ |
| Pieter Dam | 0.16 | 0.20 | **0.33** | 0.17 | 0.18 | 0.25 | 0.22 | **0.28** | 0.17 | 0.16 |  | 0.0008^*^ |
| Loskop Dam | 0.13 | 0.21 | **0.27** | 0.20 | 0.18 | 0.21 | 0.16 | 0.19 | 0.09 | 0.04 | 0.19 |  |

F*_ST_*-values < 0.05 indicate little genetic differentiation, 0.05-0.15 = moderate differentiation, 0.15-0.25 = significant differentiation, and > 0.25 = very high differentiation (given in bold) (Wright 1965). * P-values and not significant (NS) at P<0.0008
